# Study of Milkability and Its Relation With Milk Yield and Somatic Cell in Mediterranean Italian Water Buffalo

**DOI:** 10.3389/fvets.2020.00432

**Published:** 2020-08-11

**Authors:** Carlo Boselli, Massimo De Marchi, Angela Costa, Antonio Borghese

**Affiliations:** ^1^Experimental Zooprophylactic Institute Lazio and Toscana Mariano Aleandri, Rome, Italy; ^2^Department of Agronomy, Food, Natural Resources, Animals and Environment (DAFNAE), University of Padova, Legnaro, Italy; ^3^General Secretary International Buffalo Federation, Former Director Animal Production Research Institute, Rome, Italy

**Keywords:** milk flow curves, milk yield, milking time, economic approach, somatic cells

## Abstract

Milkability is defined as the ability of an animal to give a regular, complete, and rapid milk secretion by the mammary gland in response to a proper milking technique. The aim of the present study was to investigate the relationship of milkability pattern with milk yield and somatic cell score in buffaloes. Milk yield and milkability can be observed through the milk flow profiles recorded by an electronic milkmeter (Lactocorder). A total of 2,288 milk flow curves of Mediterranean Italian buffaloes were used for one-way analysis of variance, and eight milk emission patterns were studied. The most represented milk flow curve was type 3 (27.32%), followed by type 6 (17.79%) characterized by a very long plateau phase. The less represented curve was type 1 (4.41%) characterized by long lag time and low peak flow rate. According to analysis of variance, milk yield ranged from 2.21 to 5.22 kg per milking for types 1 and 6, respectively, whereas the peak flow rate was minimum (0.50 kg/min) and maximum (1.73 kg/min) for types 1 and 4, respectively. The total milking time was on average 11.29 ± 3.68 min; lag time and milk emission time averaged 2.19 ± 2.34 min and 4.30 ± 2.33 min, respectively. The 12.5% (*n* = 286) of total curves were classified as bimodal and 60 of these were found in type 4. Based on literature, type 4 curves are representative of very short teat canals and very high milk flow. Average somatic cell score was 3.63 ± 1.67 units, with maximum least-squares mean found for type 1 and minimum for type 6. Buffaloes showing curves of type 5 and 6 were characterized by the greatest milk yield at milking, lowest somatic cell score, and lowest milking time. Results of the present study evidenced that such traits could be used in the dairy buffaloes as indicators to improve udder health and milkability.

## Introduction

The buffalo population in the world was about 206 million heads in 2018, of which the 97.57% were raised in Asia (1). European buffaloes belong to the water buffalo species *Bubalus bubalis* and are farmed for dairy. Buffalo milk is an important source of energy and nutrients for the human diet in some countries, owing to a particularly high fat content (7–8%) compared with bovine milk (2). In Italy, the Mozzarella di Bufala Campana cheese is one of the most important Protected Designation of Origin foods in terms of internal market demand and export.

Monitoring the milkability of animals allows to improve efficiency of milking procedures and reduce farm production costs (3, 4). More than 50% of the working time in a dairy farm is required for milking and thus has a great impact on the farmers' profit (5, 6). To optimize milking time, farmers should select the most adequate animals or group animals with similar characteristics in terms of milkability. Having buffaloes with good milking properties is crucial for an optimal milking process, such as start milking, milk flow, uninterrupted milk flow curve, and duration of milking.

Milk production and milk flow profiles are important parameters to be recorded and evaluated, as may be informative of milking management (7). Graphical representation in milk ejection is visible through flow curves, which differ according to the dairy species (buffalo, cattle, sheep, goat, donkey, etc.) (7).

The portable milkmeter Lactocorder (WMB, Balgach, Switzerland) is normally used to record milk flow curve parameters. The latter have been well-studied in cattle (8–10), in sheep and goat (11) and, more recently, in buffalo (12–16). Briefly, the results are graphically represented in the screen of the milkmeter in three main different phases and, eventually, a fourth one. The first phase is the “milk let down phase” or lag time (LT), represented by the time elapsed between the attachment of the milking clusters and the time until there is a constant milk flow. The second one is called “plateau phase” (PPT), where the milk flow is constant. The third phase is the “decreasing phase” (DPT) that represents the time from the PPT until milk flow drops below 0.20 kg/min. An eventual fourth phase may be the “blind phase” (BT). The buffalo is often exposed to a long period of vacuum without any ejection of milk and sometimes a preliminary curve represents the cisternal fraction milk flow; this phase belongs to the LT. The BT (flow < 0.20 kg/min) occurs between the end of the DPT and the manual or automatic detachment of the milking group (both manual and automatic). Usually, the detachment is not performed promptly in order to collect the small amount of milk, the residual fraction, by stripping (often obtained by manual traction of the milking group by a milker) followed by a further overmilking before detachment of milking cluster.

Because of the udder anatomy and arrangement of the mammary tissue, cisternal fraction of milk and teat canal length are quite different in buffaloes compared with dairy cattle (13).

Milk flow curves are influenced by anatomical and physiological aspects (13, 17, 18), management (19, 20), and udder health status (12, 19, 21). Compared with bovines, buffaloes are characterized by a longer teat with a longer teat canal and by stronger muscular resistance of the teat wall. Therefore, a higher vacuum level is needed for opening the teat canal and starting milk ejection in this specie (7, 13, 18, 22). In buffalo routine management, the set-up of milking devices is a critical point and the characteristics of milking vacuum and milking pulsations are strictly connected with milk flow observations (18, 20, 22). It is well-established that udder health influences milk composition and milk flow parameters in dairy species, including buffalo (23, 24). In buffalo, as in other dairy species, the milk somatic cell count (SCC) is the most adopted indicator for udder health and mastitis (12, 23–25). Overall, in healthy conditions, buffaloes show lower milk SCC compared with bovines. The level of SCC is influenced mostly by environmental factors, in particular milking practices milking practices and farm hygiene. The aim of this study was to estimate the difference among milk flow curve types in terms of milk yield, SCC, and milkability traits in Mediterranean buffaloes in Italy.

## Materials and Methods

### Animal and Herds

This study was carried out on 2,419 Mediterranean Italian water buffaloes from 187 herds of Latium Region (Central Italy). Data were collected from 2006 to 2019. Vacuum level recorded by a milking room vacuometer was set on average at 43.4 ± 4.2 kPa, ranging from 37.0 to 54.0 kPa. Among the 2,419 recorded milk flow curves, 131 (5.41%) were discarded as they did not reach the minimum flow necessary to return the main parameters. Therefore, 2,288 milk flow curves were available, of which 779 belonged to primiparous buffaloes with average days in milk (DIM) equal to 143, and 1,401 belonged to multiparous buffaloes with average DIM equal to 124. Milk yield per milking and milk flow traits were recorded with Lactocorder portable milkmeter (26). The pre-milking routine adopted was the standard, thus including teat cleaning, short massage, and subsequent drying. In all herds, the milking cluster was applied after pre-milking routine, whereas milking cluster detachment was manually removed even if automatic takeoffs were not present or not used; only in a few cases the use of automatic detachment was activated by the milker after the beginning of the alveolar milk emission.

### Milk Flow Measurement Apparatus

The Lactocorder milkmeter (WMB, Balgach, Switzerland) starts recording information at the first teat cup attachment and finishes at the last teat cup detachment. The milkmeter was vertically attached to every milking place and inserted between the milking unit and milk pipeline by milk hose in the long milk tube. The measuring chamber consisted of one transmitting electrode and 60 vertically arranged electrodes. The evaluation of milk flow curve types was made by specific software available (LactoPro; WMB). The milk flow curve parameters and MY were recorded and a milk sample representative of the whole milking was automatically collected. For each buffalo, the device recorded 11 variables:

- MY (kg): total milk yield per head per milking from the beginning to the end of the mechanical milking;
- LT (min): lag time from the beginning of measurement until a 0.50 kg/min threshold in the milk flow was reached;
- MET (min): milk ejection time as time from milk flow rate >0.50 kg/min until milk flow decreases below 0.20 kg/min;
- PPT (min): plateau phase, as duration of plateau phase from the vertex of the incline phase to the vertex of the decline phase;
- DPT (min): time of decline phase, a period of milk flow from end of PPT to flow rate < 0.20 kg/min at the end of milking;
- T400 (min): time to get the milk flow descending from 0.40 to 0.20 kg/min;
- BT (min): time of mechanical overmilking, recorded if present at the end of DPT, with milk flow < 0.20 kg/min;
- PFR (kg/min): peak flow rate in the main milking process within a time interval of eight measuring points measured within a time interval of 22.4 s;
- AFR (kg/min): average milk flow rate during milk ejection time;
- TMT (min): total milking time, that is, the time elapsed beginning the attachment of milking cluster at mammary gland and the detachment at end of milking, including stripping time and overmilking time after stripping if they are present;
- BIMO (%): bimodality of milk ejection, that is, delayed milk ejection at the start of milking (result of the interruption of the flow at the start of milking when cisternal milk is finished and alveolar milk is still not available) as a result of improper or absence of pre-stimulation of buffalo.

### Milk Flow Curve Classification

**Figure 1:**
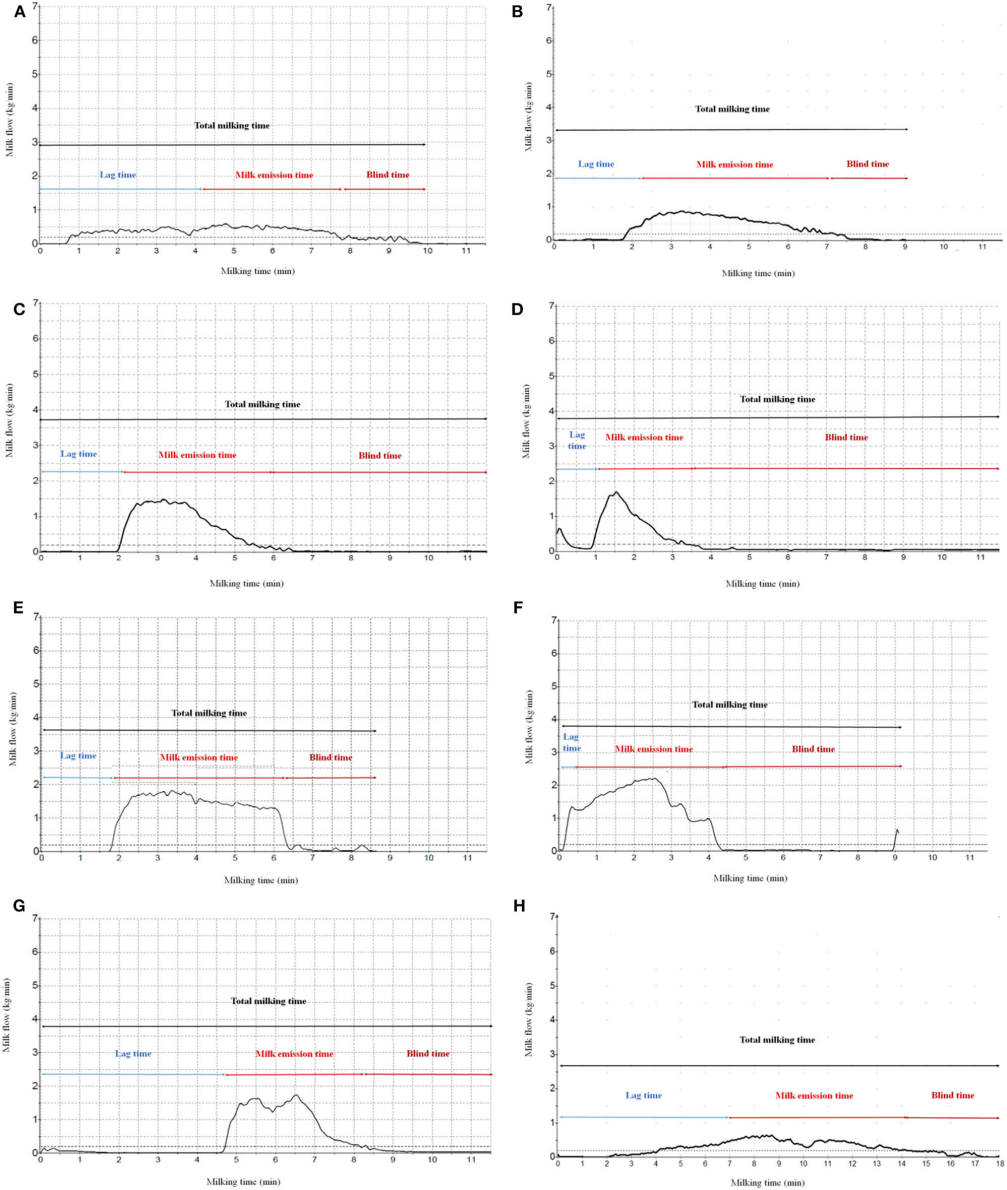
Examples of **(A)** type 1 (very low milk flow and long milking time), **(B)** type 2 (very long and continuous decreasing phase time), **(C)** type 3 (similar time between plateau and decreasing phase), **(D)** type 4 (“triangular shape,” high prevalence of bimodal curves), **(E)** type 5 (“rectangular shape,” predominant plateau phase), **(F)** type 6 (predominant plateau phase and discontinuous decreasing phase), **(G)** type 7 (double-profile and evident disturbance of milk ejection during milking), and **(H)** type 8 (very long increase phase) milk flow curve.

The classification of curves was based on a visual inspection of shape of curve, MY, and milking flow parameters. Based on their appearance, the 2,288 milk flow curves were divided into 8 types (Figure 1) as:

- Type 1: with very low milk flow and long TMT;
- Type 2: with very long and continuous DPT;
- Type 3: with similar time between PPT and DPT;
- Type 4: triangular shape, with high prevalence of bimodal curves;
- Type 5: rectangular shape, with predominant PPT;
- Type 6: with predominant PPT and discontinuous DPT;
- Type 7: with double profile with evident disturbance of milk ejection during milking;
- Type 8: with a very long LT resulting from the slow release of oxytocin and higher resistance to opening of teat canals.

### Somatic Cell Count Analysis

After collection, milk samples were refrigerated (5°C) and transferred to the milk laboratory of Istituto Zooprofilattico Sperimentale del Lazio e Toscana (Rome, Italy). The SCC determination was performed within 24 h from collection using a fluoro-opto-metric device (Fossomatic 5000 series; Foss Electric, Hillerød, Denmark). To achieve a normal distrbution of data, a logarithmic transformation was applied to SCC (27) and the somatic cell score (SCS, units) was obtained.

### Statistical and Data Analysis

The effect of milk flow curve type was estimated on all milk flow parameters, MY, and SCS through one-way ANOVA in SPSS software (version 17.0; SPSS, Chicago, IL, USA). Differences in least-squares means were tested with Bonferroni *t*-test at *p* < 0.05.

## Results

### Data Overview

The distribution of different types of milk flow curves is reported in Table 1. In both primiparous and ultiparous animals, the most represented curve was type 3 (Table 1), followed by type 6. The less represented one was type 1 (4.41%; Table 1). Milk flow curves with a gradual DPT (type 6) or continuous DPT (normal as type 2 or long as type 3) are represented in Figure 1. Triangular (type 4) and rectangular (type 5) shapes were characterized by short and long PPT, respectively, and longer and shorter DPT, respectively. Milk flow curves with a difficulty to milk release, resulting from an evident disturbance during mechanical milking, were classified in type 1 (long mechanical milking time), type 7 (double milk profile), and type 8. The trapezoidal shapes (types 2, 3, 6) are the most represented (59.53%), in comparison with rectangular shape or ideal curve (type 5, 10.62%) and the triangular shape (type 4, 9.48%). The curves with difficulties on milk emission (types 1, 7, 8) represented an important share (20.37%) of the total curves. The frequencies of the milk flow curve type were similar in primiparous and multiparous animals (Table 1).

**Table 1:** Frequency of records (%) for the 8 types of curve defined.

**Milk flow curve type**	**Whole dataset**	**Primiparous (*n* = 779)**	**Multiparous (*n* = 1,401)**
1	4.41	5.01	4.14
2	14.42	13.48	14.49
3	27.32	30.42	25.70
4	9.48	8.99	9.85
5	10.62	10.01	10.78
6	17.79	17.20	18.70
7	6.08	5.13	6.28
8	9.88	9.76	10.06

Descriptive statistics of MY, SCS, and milk flow traits are presented in Table 2; MY averaged 3.95 ± 1.86 kg. In particular, primiparous and multiparous buffaloes showed an average MY of 3.66 ± 1.72 and 4.08 ± 1.89 kg, respectively. The total milking time was on average 11.29 min per milking; MET was on average 4.30 min, with a PPT:DPT ratio equal to 85.45%, whereas the average BT was 3.76 min. Means of PFR and AFR were 1.28 and 0.82 kg/min, respectively.

**Table 2:** Overall mean (±standard deviation) and least-squares means (±standard error) of MY, milk yield per milking; BIMO, bimodality of milk ejection; PFR, peak flow rate; AFR, average milk flow rate; SCS, Somatic Cell Score; LT, lag time; MET, milk ejection time; PPT, time of plateau phase; DPT, time of decline phase; BT, time of blind phase; T400, time to get the milk flow from 0.40 to 0.20 kg/min; TMT, total milking time; in recorded curve types.

**MILK FLOW CURVE TYPES**									
**Parameter**	**1**	**2**	**3**	**4**	**5**	**6**	**7**	**8**	**Overall mean**
MY, kg	2.21 ± 0.12^f^	3.53 ± 0.10^d^^,^ ^e^	3.29 ± 0.05^e^	3.80 ± 0.13^c^^,^^d^	4.24 ± 0.11^b^^,^ ^c^	5.22 ± 0.09^a^	4.38 ± 0.17^b^	4.43 ± 0.13^b^	3.95 ± 1.86
BIMO, %	4.95 ± 2.17^b^	10.61 ± 1.69^b^	9.92 ± 1.20^b^	27.65 ± 3.04^a^	12.40 ± 2.12^b^	10.07 ± 1.49^b^	17.27 ± 3.22^b^	12.78 ± 2.23^b^	12.50 ± 33.70
PFR, kg/min	0.50 ± 0.04^f^	1.10 ± 0.03^d^	1.29 ± 0.02^c^^,^ ^e^	1.73 ± 0.05^a^	1.26 ± 0.03^c^^,^ ^e^	1.47 ± 0.03^b^	1.15 ± 0.04^d^^,^ ^e^	1.18 ± 0.04^d^^,^ ^e^	1.28 ± 0.57
AFR, kg/min	0.36 ± 0.03^e^	0.63 ± 0.01^d^	0.85 ± 0.01^b^	0.96 ± 0.03^a^	0.94 ± 0.02^a^	0.98 ± 0.02^a^	0.68 ± 0.02^c^^,^ ^d^	0.74 ± 0.02^c^	0.82 ± 0.35
SCS, units	4.12 ± 0.18^a^	3.65 ± 0.10^a^^,^ ^b^^,^ ^c^	3.79 ± 0.07^a^^,^ ^b^	3.72 ± 0.10^a^^,^ ^b^	3.46 ± 0.10^b^^,^ ^c^	3.27 ± 0.08^c^	3.63 ± 0.15^a^^,^ ^b^^,^ ^c^	3.70 ± 0.11^a^^,^ ^b^	3.63 ± 1.67
LT, min	4.09 ± 0.42^a^	2.13 ± 0.12^c^	2.08 ± 0.09^c^	2.34 ± 0.18^b^^,^ ^c^	1.95 ± 0.13^c^^,^ ^d^	1.58 ± 0.07^d^	2.31 ± 0.17^b^^,^ ^c^	2.84 ± 0.15^b^	2.19 ± 2.34
MET, min	3.00 ± 0.36^d^	4.94 ± 0.15^b^	3.35 ± 0.06^d^	3.50 ± 0.12^c^^,^ ^d^	4.05 ± 0.10^c^	5.12 ± 0.10^a^^,^ ^b^	5.73 ± 0.24^a^	5.24 ± 0.18^a^^,^ ^b^	4.30 ± 2.33
PPT, min	1.24 ± 0.19^c^	1.42 ± 0.06^c^	1.41 ± 0.03^c^	0.45 ± 0.03^d^	3.03 ± 0.09^a^	2.43 ± 0.07^b^	2.31 ± 0.17^b^	2.40 ± 0.11^b^	1.82 ± 1.50
DPT, min	1.45 ± 0.26^d^	3.27 ± 0.14^a^	1.65 ± 0.04^d^	2.52 ± 0.11^c^^,^ ^b^	0.69 ± 0.03^e^	2.37 ± 0.07^c^	2.97 ± 0.21^a^^,^ ^b^	2.34 ± 0.15^c^	2.13 ± 1.90
BT, min	1.91 ± 0.26^c^	3.50 ± 0.17^c^^,^ ^e^	4.41 ± 0.13^a^	4.26 ± 0.23^a^^,^ ^b^^,^ ^c^	4.16 ± 0.22^a^^,^ ^b^^,^ ^c^^,^ ^d^	3.48 ± 0.14^c^^,^ ^e^	2.87 ± 0.24^e^^,^ ^f^	3.27 ± 0.22^d^^,^ ^e^	3.76 ± 3.19
T400, min	0.60 ± 0.13^a^^,^ ^b^	0.87 ± 0.07^a^	0.48 ± 0.02^b^	0.45 ± 0.03^b^^,^ ^c^	0.26 ± 0.02^c^	0.53 ± 0.04^b^	0.59 ± 0.07^b^	0.54 ± 0.05^b^	0.54 ± 0.79
TMT, min	11.89 ± 0.43^a^^,^ ^b^	11.65 ± 0.19^a^^,^ ^b^	10.88 ± 0.15^b^	11.28 ± 0.27^a^^,^ ^b^	11.03 ± 0.25^b^	11.02 ± 0.16^b^	11.78 ± 0.28^a^^,^ ^b^	12.17 ± 0.25^a^	11.29 ± 3.68

*The fixed effect of milk flow curve type was significant at p < 0.001 for all traits*.

a−f *Least squares means in a row without a common superscript letter differ (p < 0.05)*.

### Patterns of Curve Types

Least-squares means (LSM) of MY ranged from 2.21 to 5.22 kg, estimated for curves in types 1 and 6, respectively. In the most frequent curve (type 3), LSM of MY was 3.29 kg. TMT ranged from 10.88 to 12.17 min, recorded for types 3 and 8, respectively. LT showed great variability, with maximum observed in type 1 (4.09 min) and minimum observed in type 6 (1.58 min). Minimum and maximum LSM of MET were 3.00 and 5.73 min, recorded for types 1 and 7, respectively. The most represented curve, type 3, showed LSM of LT and MET equal to 2.08 and 3.35 min. An opposite trend for PPT and DPT, calculated from PPT:DPT ratio, was observed in type 4 (17.86%) and type 5 (439.13%). T400 showed a maximum value in type 3 and represented on average 25.35% of the DPT (calculated between T400 and DPT ratio) and ranged from 17.85% (type 4) to 41.78% (type 1). BT ranged from 1.91 to 4.26 min, recorded in types 1 and 3, respectively. The BIMO was maximum (27.65%) in type 4 curves. Finally, LSM of SCS were from the highest (4.12 units) and the lowest (3.27 units) in types 6 and 1, respectively.

## Discussion

### Milkability Traits of Mediterranean Italian Buffalo

Findings related to MY were similar to those reported in literature for buffaloes (12, 14, 15, 19, 25, 28); however, average SCS was higher if compared at the value of 3.49, found in a field study conducted on 225 Mediterranean Italian buffaloes (21).

The TMT of this study was similar to the average values (12.90 and 11.39 min, respectively, for different vacuum levels) observed in one farm with 450 buffaloes in Italy (20) but lower to the value (6.79 min without LT) reported for buffaloes present in eight herds of Northern Italy (14).

Overall, buffaloes of this study showed a long LT (2.19 min), that is, greater compared with values available in the literature (14, 15). The longer LT before milk ejection confirmed previous studies on Mediterranean Italian buffalo, where average LT was equal to 1.94 (14) and 2.42 min (20). In Murrah buffalo (29), different values have been related to different pre-stimulation milking routine and different feeding administration during milking, with LT ranging from 1.15 to 2.57 min.

LT is influenced by the anatomical characteristics of the mammary gland and teats, with regard to resistance of teat sphincters and release of oxytocin hormone. A correct udder pre-stimulation with the simultaneous addition of a concentrate integration in the milking room has positive effects on MY and milk flow (13, 29). In fact, 1 min of manual pre-stimulation produced the best results in terms of milk ejection, AFR, PFR, and TMT.

MET was about 38.09% of the total milking time, similar to the one reported in literature (15, 19). In this time, extraction of the maximum quantity of milk is obtained. Overmilking of BT was very long (33.03% of total milking time, on average) and similar to data reported in a study on 184 buffaloes (14). In the same study, BIMO was of control group and of buffaloes treated with oxytocin were 12.40 and 8.90%, respectively.

Average AFR and PFR were 0.82 and 1.28 kg/min, confirming findings available in literature (12, 14, 16, 19, 20), with small differences between morning and evening milking and in relation with the anatomical characteristics of teats (28).

The frequencies of curve types (Table 2) resembled findings on Jersey cows (30); in this study, about 66% of curves were rectangular and descending. In a field study carried out on 497 Holstein cows (31), 59.4% of total curves were those with a gradual or continuous decrease phase, similar to the percentage (59.53%) of the present study (type 2, type 3, and type 6). In the same study (31), only 6.4% of the curves were trapezoidal (it represents the optimal shape which includes a short and steep inclination phase, a plateau phase along a short and steep decline phase), whereas in the present study, type 5, similar in shape to the trapezoidal shape, was found on 10.42%, higher than the trapezoidal one. The same researchers reported that it may be useful to consider that the goal of most farmers is to select animals with milk emission similar to trapezoidal shape of milk flow curve, an indicator of a physiological letdown of milk.

In general, in this study, the BT phase was sometimes predominant when compared with other phases (Figure 1). Presence of long BT increases management costs and mastitis risk as a result of tissue stress and may indicate suboptimal udder pre-milking stimulation. In other species, it has been demonstrated that the reduction of BT is essential to improve udder health, longevity, and milking efficiency (30, 32, 33).

### Fixed Effect of Milk Curve Type

According to the ANOVA (Table 2), LSM of MY in type 1 curve was the lowest. In fact, type 1 curve is characterized by very long TMT and thus limited MY, difficulties in LT resulting from changes in the anatomical parameters of teat canal and likely to low variation of oxytocin blood level. On the contrary, MY of type 6 was the highest; this curve is characterized by a high PFR, long PPT, and probably by different milk distribution in one or more quarters (20). Types 2 and 3 curves (36.8% of total curves) were characterized by intermediate MY, with different lengths of DPT; this phase, where the milk flow is decreasing and continuous, is predominant with respect to PPT. At the udder level, the DPT mainly begins when milk flow from one or more quarters stopped. In contrast, at the quarter level, DPT starts when cisternal filling by alveolar milk is lower than intensity of milk removal (10). On the other hand, a short phase of decline is desirable to ensure the machine's adhesion time and milking stress within limits. A long decline is generally an indicator of an overmilking of one or more quarters. However, about 15–20% of curves for cattle were reported with a medium-long physiological DPT with a constant reduction of milk flow (26).

Type 4 curve is called “triangular shape” because it shows a very high PFR, short PPT, and long DPT; the BIMO was the highest in this type (Table 2). A similar type was found in high percentage (40% of total curves) in dairy dromedary camel (34), characterized by higher peak flow levels and short milking durations which depend mainly on the amount of milk stored in the udder.

Types 7 and 8 (15.95% of total curves) showed evident difficulty of milk emission with similar MY (4.38 vs. 4.43 kg, respectively; Table 2). In type 7, the double profile (Figure 1) is probably caused by an evident disturbance of milk ejection, related to blood oxytocin concentration changes, or to position changes of milking cluster during MET (15). Type 8 is characterized by a slight—but continuous—increase of milk ejection, probably as a result of a slow increase of blood oxytocin concentration and higher resistance to opening of teat sphincters.

Finally, the ideal milk emission type was recognized in type 5 curve, the “rectangular shape,” with high PFR, AFR, and PPT:DPT ratio.

Regarding SCS, the most desirable value was found in curves of types 5 and 6; both SCC and SCS are closely related to the management of the farm, especially milking procedures (35, 36). In this paper, only data of a single farm were used and therefore the effect of type of management was minimized. In agreement with the literature (37), the greatest MY was found in correspondence of the lowest SCS (curves types 5 and 6) and vice versa; this could suggest that animals with optimal milk flow curves were characterized by less mammary gland epithelium stress and good milk flow and therefore showed favorable MY and SCS. On the other hand, it is established that suboptimal milk ejection patterns may be alert for udder infection and/or inflammation. However, the inverse trend of MY and SCS could be also partly caused by a dilution effect. In fact, in this study, there was no correction for the productivity level (genetic potential) of each buffalo. Genetically, high-producing heads are expected to be under oxidative stress, have greater susceptibility to udder issues, and overall present higher milk SCS.

## Conclusion

The aim of this study was to estimate the differences among on milk yield, SCC, and milk flow traits recorded by a portable milkmeter in Mediterranean Italian buffaloes. We analyzed milk flow curves coming from more than 2000 Mediterranean Italian buffalo, and then we proposed a classification to highlight some significant difference among curves that could have an impact on milk production and udder health. Results highlighted a high prevalence of overmilking that may be responsible for the adoption of higher TMT in buffalo compared with cattle. In general, it is recommended to apply adequate udder stimulation before milking to reduce LT, increase AFR, and limit TMT. Moreover, adequate milking practices would translate into decrease of working time and improvement of farmer's income through better milk quality and less udder issues. The results of this study have allowed to identify optimal milk flow curve types in terms of milk production and udder health; such findings might be useful to buffalo breeders' associations to address farmers' management and also to define potential new breeding objectives. Further research is needed to investigate the variability of such phenotypes at the population level and understand if they may be exploited as indicator traits for breeding purposes.

## Data Availability Statement

The datasets presented in this article are not readily available because data that support the findings of this study are available at the corresponding author, [CB], upon reasonable request. Requests to access the datasets should be directed to carlo.boselli@izslt.it.

## Ethics Statement

Ethical approval was not required for the present study, since we recorded only milking data during mechanical milking. Written informed consent for participation was not obtained from the farmers because collection and recording of data was carried out during official controls in milking parlour.

## Author Contributions

CB and AB designed and carried out the surveys in the milking parlour. CB processed the data. CB and AC discussed the results and wrote the draft. Finally, all authors revised the manuscript, contributed to the article and approved the submitted version.

## Conflict of Interest

The authors declare that the research was conducted in the absence of any commercial or financial relationships that could be construed as a potential conflict of interest.
